# A Systematic Review to Evaluate the Association between Clean Cooking Technologies and Time Use in Low- and Middle-Income Countries

**DOI:** 10.3390/ijerph16132277

**Published:** 2019-06-27

**Authors:** Suzanne M. Simkovich, Kendra N. Williams, Suzanne Pollard, David Dowdy, Sheela Sinharoy, Thomas F. Clasen, Elisa Puzzolo, William Checkley

**Affiliations:** 1 Division of Pulmonary and Critical Care, School of Medicine, Johns Hopkins University, Baltimore, MD 21287, USA; 2 Department of Epidemiology, Bloomberg School of Public Health, Johns Hopkins University, Baltimore, MD 21205, USA; 3 Department of Environmental Health, Rollins School of Public Health, Emory University, Atlanta, GA 30322, USA; 4 Department of Public Health and Policy, The University of Liverpool, Liverpool L69 3BX, UK

**Keywords:** Cooking, air pollution, time, wage, biomass, stoves

## Abstract

Interventions implementing clean fuels to mitigate household air pollution in low- and middle-income countries have focused on environmental and health outcomes, but few have evaluated time savings. We performed a systematic review, searching for studies of clean fuel interventions that measured time use. A total of 868 manuscripts were identified that met the search criteria, but only 2 met the inclusion criteria. Both were cross-sectional and were conducted in rural India. The first surveyed the female head of household (141 using biogas and 58 using biomass) and reported 1.2 h saved per day collecting fuel and 0.7 h saved cooking, resulting in a combined 28.9 days saved over an entire year. The second surveyed the head of household (37 using biogas and 68 using biomass, 13% female) and reported 1.5 h saved per day collecting fuel, or 22.8 days saved over a year. Based on these time savings, we estimated that clean fuel use could result in a 3.8% or 4.7% increase in daily income, respectively, not including time or costs for fuel procurement. Clean fuel interventions could save users time and money. Few studies have evaluated this potential benefit, suggesting that prospective studies or randomized controlled trials are needed to adequately measure gains.

## 1. Introduction

Forty percent of households in low- and/or middle-income countries (LMICs) use biomass fuels as their primary fuel for cooking and heating [1]. Biomass burning results in household air pollution (HAP), which is composed of harmful pollutants such as fine particulate matter (PM_2.5_), nitrous oxide, endotoxins, and carbon dioxide [2]. Burning biomass fuel for cooking also has negative environmental consequences, exacerbating global warming and contributing to deforestation [3]. Exposure to HAP has been associated with numerous health problems, including chronic obstructive pulmonary disease, childhood pneumonia, lung cancer, head and neck cancer, hypertension, and cataracts [4]. In 2017, HAP exposure was associated with 1.6 million premature deaths and 59.5 million disability-adjusted life-years lost worldwide [5].

Exposure to HAP is also associated with welfare and labor income losses estimated at USD $1.6 trillion and USD $94 billion, respectively [6,7]. Cooking with biomass requires that people spend time collecting fuel (often over long distances), preparing the fuel by drying and cutting or shaping it, and igniting and tending the fire. This lost time, along with the poor health caused by the smoky environment, diminishes productivity and opportunity for income generation. Women and children face the greatest opportunity cost from time spent collecting fuel and cooking, given that they are most often responsible for these tasks in LMICs [1,8]. Collecting fuel and cooking limits the time available to obtain an education, earn income, participate in nonpaid work activities, or relax [1,9,10].

Access to and sustained use of clean cooking technologies could reduce the time required for collecting fuel and cooking. However, few clean cooking promotion efforts have documented the amount of time saved by users of clean cooking technologies as a potential benefit. As the cost of fuel for clean energy is one of the biggest barriers to adoption, time savings could potentially offset this cost.

Prior studies that have evaluated the amount of time spent collecting fuel and cooking have focused on improved biomass-burning stoves [11,12]. However, mounting evidence has suggested that improved biomass-burning stoves are not able to reduce household annual mean concentrations of fine particulate matter below the World Health Organization’s intermediate target [13]. As a result, policy-makers are shifting to promote clean-burning technologies such as liquefied petroleum gas (LPG), ethanol, electricity, biogas, and solar energy to achieve health benefits [14,15]. Therefore, there is a need to document the extent to which clean fuels can also achieve time savings for the household, in addition to the more widely recognized potential health and environmental impacts. To answer this question, we performed a systematic review to identify existing studies that compared biomass and clean fuel users in terms of time spent collecting fuel and cooking. We also estimated the potential user-level economic impact of time savings from clean fuel adoption.

## 2. Methods

### 2.1. Search Strategy and Study Selection

We followed the Preferred Reporting Items for Systematic Reviews and Meta-Analyses (PRISMA) statement for the preparation of this systematic review [16]. The first author (S.S.) and an informationist at the Johns Hopkins Welch Medical Library performed the literature review. Search terms are listed in Appendix A. The public databases searched included PubMed, EMBASE, Web of Science, Eldis, Global Health via Ovid, Global Index Medicus via the World Health Organization, and the Virtual Health Library. The World Bank report “Household Cooking Fuel Choice and Adoption of Improved Cookstoves In Developing Countries”, which evaluated the literature on clean cooking interventions through June 2014, was also reviewed for manuscripts meeting the search criteria [17]. Our search included reports, research abstracts from meeting proceedings, and unpublished studies. The search period was 1 January 1950 to 1 April 2018. Titles and abstracts of all identified manuscripts were reviewed for eligibility. All manuscripts that met the eligibility criteria were obtained and reviewed in full. References from identified studies were hand-searched for any additional relevant studies for analysis.

### 2.2. Study Eligibility

We list study eligibility criteria in Table 1. An LMIC is defined as a country whose gross national income per capita in 2017 using the World Bank Atlas method was less than USD $12,055 [18]. Biomass fuels were defined as firewood, dung, agricultural crop waste, coal, and charcoal, and clean fuels as LPG, biogas, ethanol, electricity, pellets, and solar power. Two authors (Suzanne M. Simkovich and K.W.) independently reviewed all studies for eligibility. Both authors’ selections were compared, and disagreements were resolved through discussion. A third author (W.C.) reviewed the selected studies and agreed with the study selection.

### 2.3. Risk of Bias and Quality Assessment

Studies that met the eligibility criteria were evaluated for quality objectively using the modified Newcastle–Ottawa Scale (NOS) for assessing the quality of cross-sectional studies in meta-analyses and subjectively by the authors [19,20]. An evaluation of each selected study was completed by Suzanne M. Simkovich. and K.W. using this scale. Subjectively, each study was reviewed for quality according to the following standards: (1) A robust study design to ensure quality evidence (randomized controlled, cohort, case-controlled, or cross-sectional studies), (2) a biomass-using comparison group, and (3) quantitative outcomes. These criteria were measured based on whether they were present or not.

### 2.4. Time Collecting Fuel and Cooking

Time spent collecting fuel and cooking was abstracted and recorded directly from each study. All abstracted time was adjusted to tenths of hours collecting fuel and tenths of hours cooking per day. Differences between the biomass and clean fuel groups were defined as time savings in hours per day.

### 2.5. Shadow Wages

A shadow wage is the amount of money a participant in each study could be expected to earn if the opportunity to work were available in his or her location. To determine these wages, we reviewed government and private sector sources along with the literature. We decided to use the agricultural sector wage rates published by the Indian government, which summarize average wages based on a bimonthly survey of workers and report after-tax wages by sex, profession, and specific location.

### 2.6. Time Valuation

We applied a shadow wage to the time saved collecting fuel and cooking based on the government-reported rates for each study site. We followed the guidelines from Whittington and Cook’s review on valuing time in LMICs, valuing time at 50% of after-tax wages [21]. Our overall calculations assumed that 50% of time saved would be used for income generation. Given that all time savings may not be used for this purpose, we also conducted sensitivity analyses to understand how estimates would change if 25%, 75%, and 100% of time saved were spent on income generation [21]. We assumed all increased income from time saved is paid in currency and not in-kind exchanges. Shadow wages were converted to USD using the United Nations 2016 average rates [22,23].

## 3. Results

### 3.1. Characteristics of Selected Studies

**Figure 1:**
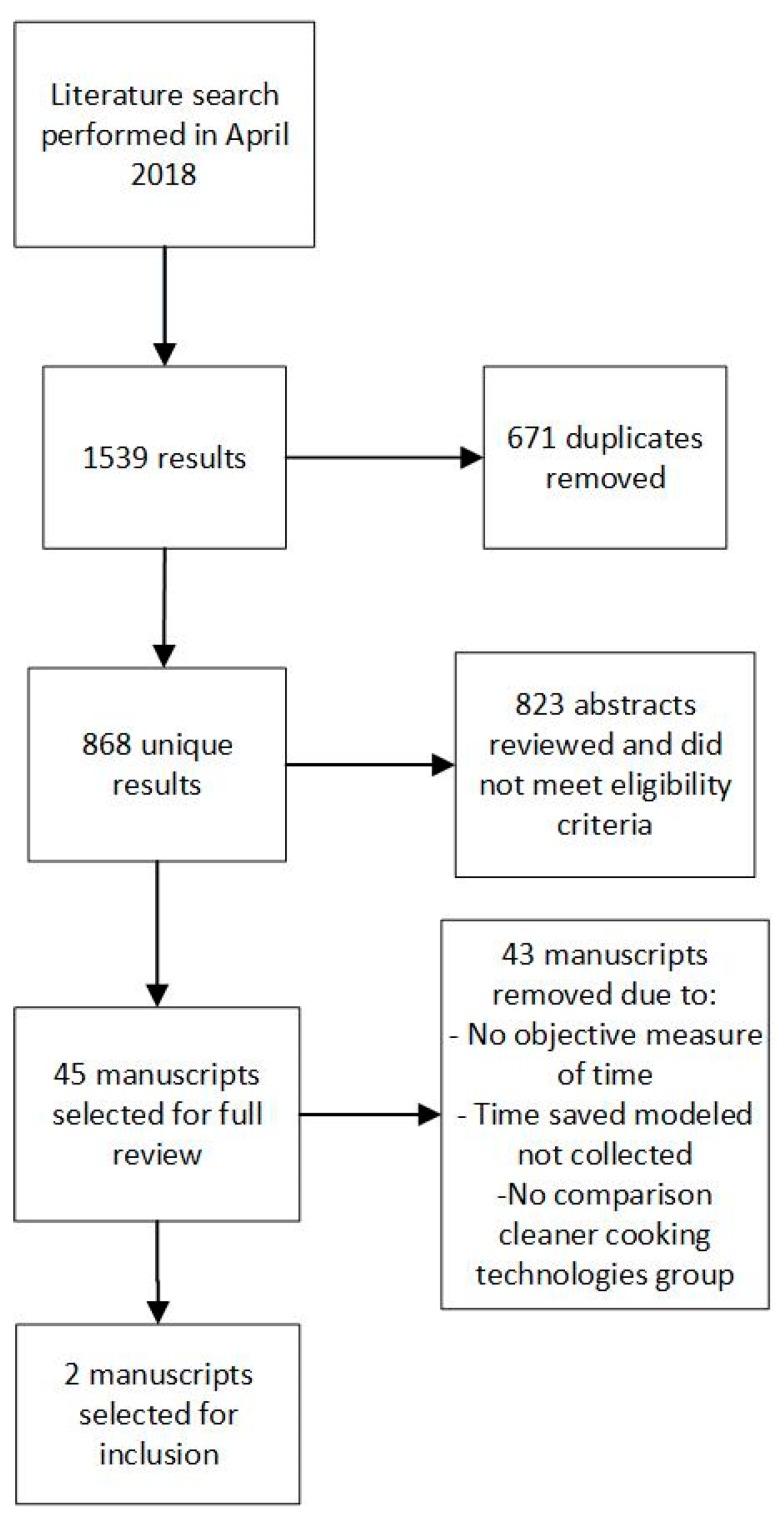
Preferred Reporting Items for Systematic Reviews and Meta-Analyses (PRISMA) diagram.

We identified 1539 manuscripts through the initial literature search. After removing 671 duplicates, the search yielded 868 unique results. Titles and abstracts that were not related to the subject were removed, resulting in 45 manuscripts. The full text of these 45 manuscripts were reviewed, resulting in two manuscripts that met the inclusion criteria and quality standards (Figure 1). No additional manuscripts or reports were identified through reviews of the gray literature or citations of selected papers.

A total of 304 participating households were enrolled in the two included studies. Both Anderman et al. [24] and Lewis et al. [25] were conducted in rural, agricultural-based settings in India. Time collecting fuel and cooking were abstracted from each study and are summarized in Table 2. Both studies reported time saved in fuel collection by cooking with clean fuels. Anderman et al. reported time saved cooking, whereas Lewis et al. did not evaluate this outcome [24,25]. Annualized, Anderman et al. reported an average of 28.9 days saved from reduced time spent collecting fuel and cooking, and Lewis et al. reported an average of 22.8 days saved from reduced time spent collecting fuel.

We summarize the findings of each study in the following two paragraphs. The first study (Anderman et al.) found that biogas-using participants spent 1.2 fewer hours collecting fuel and 0.7 fewer hours cooking per day than participants using biomass fuels [24]. This cross-sectional study was conducted in 2013 in the state of Karnataka, India. One-hundred and ninety-nine randomly selected households (141 households using biogas stoves and 58 households using biomass) were sampled. The biogas stoves were built as part of the Bagepalli clean development mechanism, and the stoves were provided for free. The comparison group was selected from households in villages near the intervention households that met the criteria for a biodigester (i.e., owned a cow and had sufficient yard space) but could not install biodigesters given the rocky terrain. The comparison group primarily used wood stoves for cooking, except five households that used a kerosene stove. Time was evaluated through a survey completed with the female head-of-household, in which she was asked to recall her activities over the previous 24 h. The study did not indicate whether time estimates for fuel collection included time spent collecting dung to feed the biodigester in the treatment group, nor whether any costs were involved in obtaining fuel (i.e., to purchase it or pay for transportation to collect it). The manuscript did not indicate whether other household members spent additional time collecting fuel or cooking beyond that reported by the female head-of-household. The researchers indicated that two households in the treatment group used a wood stove concurrently with their biogas stove, but no objective stove use monitoring devices were used [24].

The second study (Lewis et al.) found that participants using clean fuel (biogas, LPG, or electricity) spent 1.5 fewer hours collecting fuel per day than participants using biomass fuels [25]. Time spent cooking was not measured. This was a cross-sectional study conducted in 2011–2012 in the state of Odisha, India. One-hundred and five households were sampled across nine villages (68 households used either biogas, LPG, and/or electricity and 37 households used biomass). The Odisha Renewable Energy Development Agency had promoted and installed fixed-dome household biogas plants free of charge to households. Biomass fuels used included wood, dung, twigs, and crop residue. Clean fuel users had higher education and socioeconomic statuses than biomass users, so these variables were controlled for in the analyses. Surveys were completed with the head-of-household (13% female) with input from the primary cook: Participants were asked to recall how much time all household members spent gathering firewood in the previous 24 h. This study did not indicate whether fuel collection estimates included time spent collecting dung for biogas plants or for obtaining LPG refills. The manuscript also did not indicate the costs involved in purchasing biomass or clean fuels, if any. None of the households with clean fuel stoves used them exclusively, with only two households reporting using a traditional stove for less than 1 h during the previous 24 h. Stove use was assessed through self-reporting: No stove use monitoring temperature sensors were used [25].

### 3.2. Quality of Included Studies

The two included manuscripts met subjective quality standards by the authors. The results of the NOS are presented in Table A1 of the Appendix B. All studies received a quality rating of at least six out of nine possible stars on the NOS.

### 3.3. Heterogeneity in Included Studies 

The two studies had several differences. First, the types of clean fuels and biomass were slightly different across studies. Anderman et al. compared biogas fuel users to users of biomass and kerosene [24]. Lewis et al. grouped biogas, LPG, and electric stove users into a clean stove user group, which was compared to biomass users [25]. The surveyed respondents also differed, with Anderman et al. surveying the female household head and Lewis et al. surveying the head of household with input from the primary cook [24,25]. In both studies, it was unclear whether the respondent was primarily responsible for collecting and preparing fuel or cooking [24,25]. The time estimates from Anderman et al. focused on the time spent by the female head-of-household, while the time estimates in Lewis et al. were at the household level [24,25]. Stove stacking, or concurrent use of biomass and clean fuel, among clean fuel participants was much higher in Lewis et al. than in Anderman et al [24,25]. To assess stove use, Lewis et al. relied on self-reporting, while Anderman et al. did not describe in detail how concurrent firewood and biogas was assessed [24,25].

### 3.4. Economic Impact of Time Savings to a Household

**Figure 2:**
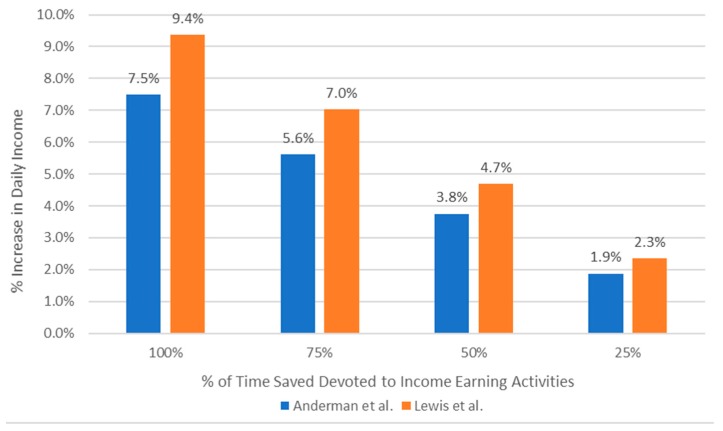
Potential increase in income by percentage of time devoted to income earning. This chart shows the potential percentage increase in income (*y* axis) a user in each study could potentially earn by the amount of time saved devoted to income-earning activities (*x* axis). Time is valued at 50% of local after-tax wages for each study.

Anderman et al. and Lewis et al. both found time savings among clean fuel users compared to biomass stove users [24,25]. The Indian government average daily after-tax wages for female field laborers was USD $3.65 in Karnataka, India (Anderman et al.), and USD $2.58 in Odisha, India (Lewis et al.), based on an eight-hour day. [24,25] Using data from Anderman et al., we estimated that there would be a 3.8% increase in daily income if 50% of the time saved from reduced fuel collection were devoted to income-earning activities. Furthermore, if 50% of the time saved cooking were devoted to income-earning activities, this would result in an additional 2.2% increase in income, for a combined increase of 5% in daily income. Using data from Lewis et al., we estimated that there would be a 4.7% increase in daily income if 50% of the time saved collecting fuel were devoted to income-earning activities. We show potential increases in income if varying amounts of time savings were used for income generation in Figure 2, based on the time savings estimates from each study. These estimates do not account for the costs of fuel or the costs of delivery or transportation for clean fuel refills.

## 4. Discussion

Time savings may be an important and measurable benefit of climbing the energy ladder in households in LMICs. However, there is a dearth of information in the published literature about time saved by women or household members who use clean fuels when compared to biomass fuels. Indeed, our systematic review only identified two studies between 1950 and 2018 that collected analyzable data. During our search, we found several studies that evaluated time savings but did not meet our inclusion criteria because there was not a quantitative measure of time use, time was modeled based on assumptions and not collected directly from participants, or the evaluation did not compare a clean fuel group to a biomass fuel group. The low number of manuscripts identified shows an important gap in the published literature and the need for increased prospective studies or randomized controlled trials to better quantify time savings.

Nonetheless, both studies found that users of clean fuels spent considerably less time collecting fuel and cooking compared to biomass users. Based on time saved, our models indicate that daily wages could be increased by 3.8–4.7% by using clean cooking technologies. It is likely that the estimates of time savings from these two studies are conservative given that exclusive use of clean fuels was not consistent. Under conditions of exclusive clean fuel use, time savings may be more substantial than reported here.

Other studies have suggested that time savings from switching from biomass to cleaner fuels could be considerable [26,27]. Unfortunately, these studies did not use designs or methods that allowed for adequate quantification of time savings from clean fuel adoption. Malla et al. estimated cooking times by measuring the amount of time carbon monoxide exceeded 9 ppm to indicate biomass stove use and stove-use temperature monitors indicated LPG stove use. They estimated that LPG users spent 1.0 fewer hours per day cooking than biomass users [28]. Studies on improved biomass stoves have also shown a decrease in time spent collecting fuel and cooking. A study in Senegal reported an aggregate reduction in time spent collecting fuel by 2.5 h per week, which was associated with the use of an improved woodburning stove [11]. A study in Malawi found that participants using a Philips improved wood-burning fan-assisted stove reduced daily fuel collection time by 31 min per day and cooking time by 2.9 h per day compared to traditional biomass stove users [12].

Findings in the nonpeer-reviewed literature were consistent with those of our systematic review. In the Energy Sector Management Assistance Program 2004, a survey conducted in states across India, users of LPG self-reported spending less time collecting fuel each day when compared to those who used biomass [29]. A cross-sectional survey conducted in Indonesia in 2015 (which lacked comparable biomass and clean fuel groups) indicated that LPG could reduce time spent cooking the main meal by 22 min compared to biomass [30]. A recently published report by the World Bank using data from Rwanda found that households spent an average of seven hours per week collecting fuel. Respondents reported that the ability of clean fuel to reduce fuel collection times was one of the main reasons why they would be willing to pay for clean fuel [31].

This systematic review had several strengths. We included databases across multiple disciplines to encompass potential evaluations done from different perspectives, including economics, behavioral sciences, finance, health, engineering, chemistry, and environmental science. To further expand our search, we searched the gray literature and evaluated reports from nonprofit organizations for evidence of time savings. We included studies from all LMICs and did not limit our search based on language unless a translation could not be done.

We also recognize that our systematic review had some shortcomings. First, this evaluation included only two studies, which is too small of a sample to draw any firm conclusion regarding time savings. Furthermore, these studies were cross-sectional and were not a result of randomized controlled trials. The decision not to include improved biomass stoves, which have not been effective in lowering PM_2.5_ to World Health Organization recommended levels, significantly decreased our sample size of included studies [32]. Second, we only found studies that were conducted in rural India, where time use patterns may be different than in other areas of the world. It was also not clear whether the member of the household surveyed was the primary person responsible for collecting fuel or cooking or whether the respondent accounted for the time of all household members or just him/herself. The manuscripts did not indicate whether any of the time savings were from reduced fuel collection or cooking by children, which could be used for education instead of income generation. A 1-day, 24-h recall period is not ideal, as patterns of time use could change over the course of a week and could be seasonally dependent. Additionally, many other opportunities for time savings were not accounted for in this analysis, such as time savings or increased productivity from reduced illness and less time spent caring for ill household members due to a less smoky environment [33]. At the same time, our analysis did not account for time and money spent procuring clean fuels. Lastly, estimates of time use and stove use were measured through self-reported recall. More objective measures of time, such as stove use monitors that objectively record the use and time spent cooking with specific stoves, would allow for more accurate assessments.

How time is used once it is saved from switching from biomass fuel to cleaner cooking technologies is less clear. We modeled a user’s potential gain in income, but this is just one potential benefit. Time could also be used for leisure activities, housework, caregiving, or educational activities. Even if extra time is not used for income earning, clean fuels can empower households to have more control over their time. Wage determination also affected our potential increase in income for users who switched from biomass to clean cooking technologies. We used the wages reported by the government of India for agricultural workers in each specific location of the included studies [34]. We selected this source because the Indian government surveys workers in each district twice per month to obtain this data. Even with direct surveying of workers, we acknowledge this may not be accurate, as labor markets in LMICs are often not formal and transactions are conducted through in-kind exchanges of goods and bartering of services [35].

Determining the true potential economic benefit based on the two studies is difficult, as not all costs were accounted for in our evaluation. These studies did not report the amount of money and/or time that people spend obtaining and purchasing clean fuel. Some studies have suggested that time spent obtaining clean fuel can be substantial. For example, Pollard et al. reported that people in Puno, Peru, spend between 67 and 93 min exchanging their LPG tanks [36]. These time and monetary costs may offset effective increases in income and time savings and must be considered when assessing the economic impact of clean cooking technology interventions. Furthermore, realizing potential time savings and economic benefits of clean fuels requires that people adopt and consistently use clean cooking technologies, which may involve overcoming other cultural and social factors that drive household decisions related to stove use.

To document the potential benefit of time savings, more rigorous evaluations of time need to be conducted in randomized controlled trials of clean fuels. More research is needed to understand household use of time savings and the direct or indirect effects on socioeconomic status. Several studies, such as the Cardiopulmonary and Household Air Pollution (CHAP) and the Household Air Pollution Intervention Network (HAPIN) trials, are in the process of amassing more evidence on perceptions and use of time savings [37]. Instruments that can evaluate the use of time prospectively and be modified to accommodate different settings and make them comparable are needed. Technology could assist with this, for example by allowing people to tabulate their activities in real time with cellphone apps or digital watches. Policy-makers, governmental leaders, and community-level program implementers should take into account the potential time savings and associated increases in economic productivity when considering whether to implement a clean cooking technology program.

## 5. Conclusions

When evaluating clean fuel interventions, time savings to users should be considered as a potential benefit to the household or individual. Switching from biomass to clean fuel can result in significant time savings, which in turn could be used for income generation or increased leisure or education. Time savings is an outcome that has not been commonly evaluated in previous studies, and prospective studies or randomized controlled trials should include this as an outcome. Clean energy programs carry potential economic benefits for rural LMIC communities through time savings in addition to the more commonly anticipated health and environmental gains.

## Figures and Tables

**Table 1:** Eligibility criteria. LMIC: Low- and/or middle-income country.

Eligibility Criteria	Ineligibility Criteria
Study conducted in an LMIC	English translation not possible
Observational study or randomized controlled trial comparing biomass fuel users to clean fuel users in the same setting	Determined time savings without an objective measure of time (i.e., modeled based on increased speed of cooking)
Collected an objective measure of time spent cooking and/or collecting fuel (interview where time quantity was identified, quantitative questionnaire or stove use monitored)	No biomass comparison group

**Table 2:** Eligible studies.

Study	Study Design	Cohort (Type of Fuel)	*n*	Time Collecting Fuel (h/day) (s.d.)	Time Cooking (h/day) (Standard Deviation)
Anderman et al. [24]	Cross- sectional	Biomass fuel (wood and kerosene)	58	1.2 (0.9)	3.4 (0.9)
		Clean fuel (biogas)	141	0 (0.1)	2.7 (1.2)
		Time saved		1.2 (0.14)	0.7 (0.17)
Lewis et al. [25]	Cross- sectional	Biomass fuel (wood, twigs, dung, and crop residue)	68	2.9 (3.8)	N/A
		Clean fuel (biogas, liquified petroleum gas, or electricity)	37	1.4 (1.9)	
		Time saved		1.5 (0.56)	

## Appendix A. Search Terms

**PubMed**

(((clean* [tw] AND fuel*[tw]) OR (clean*[tw] AND “energy”[tw]) OR (“biogas”[tw] OR “bio gas”[tw]) AND (stove*[tw] OR cookstove*[tw] OR cooktop*[tw] OR cooking*[tw] OR “kitchen”[tw] OR “kitchens”[tw])) OR (“lpg stove”[tw] or “lpg stoves”[tw] or “gas stove”[tw] or “gas stoves”[tw] or “electric stove”[tw] or “electric stoves”[tw] OR “ethanol stove”[tw] OR “ethanol stoves”[tw] OR “ethanol burner”[tw] OR “ethanol burners”[tw] or “gas burner”[tw] or “gas burners”[tw] or “gas cooktop”[tw] or “gas cooktops”[tw] OR “household energy”[tw] OR biodigester*[tw] OR “compressed biomass”[tw] OR briquette*[tw] OR “biomass pellet stove”[tw] OR “biomass pellet stoves”[tw])) AND (“water boil time”[tw] OR “water boil times”[tw] OR “water boiling time”[tw] OR “water boiling times”[tw] OR “water boil test”[tw] OR “water boil tests”[tw] OR “water boiling test”[tw] OR “water boiling tests”[tw] OR “time factor”[tw] OR “time factors”[tw] OR “time savings”[tw] OR “times saved”[tw] OR “saved time”[tw] OR “time saved”[tw] OR “time spent”[tw] OR “time benefit”[tw] OR hour*[tw] OR minute*[tw] OR performance*[tw] OR consumption*[tw] OR “time saving”[tw])

**EMBASE**

((((clean* AND fuel*):ti,ab,kw,de OR (clean* AND ‘energy’):ti,ab,kw,de OR (‘biogas’ OR ‘bio gas’):ti,ab,kw,de AND (stove* OR cookstove* OR cooktop* OR cooking* OR ‘kitchen’ OR ‘kitchens’):ti,ab,kw,de) OR (‘lpg stove’ or ‘lpg stoves’ or ‘gas stove’ or ‘gas stoves’ or ‘electric stove’ or ‘electric stoves’ OR ‘ethanol stove’ OR ‘ethanol stoves’ OR ‘ethanol burner’ OR ‘ethanol burners’ or ‘gas burner’ or ‘gas burners’ or ‘gas cooktop’ or ‘gas cooktops’ OR ‘household energy’ OR biodigester* OR ‘compressed biomass’ OR briquette* OR ‘biomass pellet stove’ OR ‘biomass pellet stoves’):ti,ab,kw,de) AND (‘water boil time’ OR ‘water boil times’ OR ‘water boiling time’ OR ‘water boiling times’ OR ‘water boil test’ OR ‘water boil tests’ OR ‘water boiling test’ OR ‘water boiling tests’ OR ‘time factor’ OR ‘time factors’ OR ‘time savings’ OR ‘times saved’ OR ‘saved time’ OR ‘time saved’ OR ‘time spent’ OR ‘time benefit’ OR hour* OR minute* OR performance* OR consumption* OR ‘time saving’):ti,ab,kw,de

**Web of Science**

# 6

#5 AND #4 AND #3

# 5

TS = (“water boil time” OR “water boil times” OR “water boiling time” OR “water boiling times” OR “water boil test” OR “water boil tests” OR “water boiling test” OR “water boiling tests” OR “time factor” OR “time factors” OR “time savings” OR “times saved” OR “saved time” OR “time saved” OR “time spent” OR “time benefit” OR hour* OR minute* OR performance* OR consumption* OR “time saving”)

# 4

TS = (“lpg stove” or “lpg stoves” or “gas stove” or “gas stoves” or “electric stove” or “electric stoves” OR “ethanol stove” OR “ethanol stoves” OR “ethanol burner” OR “ethanol burners” or “gas burner” or “gas burners” or “gas cooktop” or “gas cooktops” OR “household energy” OR biodigester* OR “compressed biomass” OR briquette* OR “biomass pellet stove” OR “biomass pellet stoves”)

# 3

#2 AND #1

# 2

TS = (stove* OR cookstove* OR cooktop* OR cooking* OR “kitchen” OR “kitchens”)

# 1

TS = (clean* AND fuel*) OR TS=(clean* AND “energy”) OR TS=(“biogas” OR “bio gas”)

**ELDIS**

Must be browsed independently (http://bit.ly/2oIh7ZN).

**Global Health via Ovid**

((clean* AND fuel*) OR (clean* AND “energy”) OR (“biogas” OR “bio gas”) AND (stove* OR cookstove* OR cooktop* OR cooking* OR “kitchen” OR “kitchens”)) OR (“lpg stove” or “lpg stoves” or “gas stove” or “gas stoves” or “electric stove” or “electric stoves” OR “ethanol stove” OR “ethanol stoves” OR “ethanol burner” OR “ethanol burners” or “gas burner” or “gas burners” or “gas cooktop” or “gas cooktops” OR “household energy” OR biodigester* OR “compressed biomass” OR briquette* OR “biomass pellet stove” OR “biomass pellet stoves”)) AND (“water boil time” OR “water boil times” OR “water boiling time” OR “water boiling times” OR “water boil test” OR “water boil tests” OR “water boiling test” OR “water boiling tests” OR “time factor” OR “time factors” OR “time savings” OR “times saved” OR “saved time” OR “time saved” OR “time spent” OR “time benefit” OR hour* OR minute* OR performance* OR consumption* OR “time saving”)

**Global Index Medicus**

((((clean* AND fuel*) OR (clean* AND “energy”) OR (“biogas” OR “bio gas”) AND (stove* OR cookstove* OR cooktop* OR cooking* OR “kitchen” OR “kitchens”)) OR (“lpg stove” or “lpg stoves” or “gas stove” or “gas stoves” or “electric stove” or “electric stoves” OR “ethanol stove” OR “ethanol stoves” OR “ethanol burner” OR “ethanol burners” or “gas burner” or “gas burners” or “gas cooktop” or “gas cooktops” OR “household energy” OR biodigester* OR “compressed biomass” OR briquette* OR “biomass pellet stove” OR “biomass pellet stoves”)) AND (“water boil time” OR “water boil times” OR “water boiling time” OR “water boiling times” OR “water boil test” OR “water boil tests” OR “water boiling test” OR “water boiling tests” OR “time factor” OR “time factors” OR “time savings” OR “times saved” OR “saved time” OR “time saved” OR “time spent” OR “time benefit” OR hour* OR minute* OR performance* OR consumption* OR “time saving”)

**VHL Regional Portal**

(((clean* AND fuel*) OR (clean* AND “energy”) OR (“biogas” OR “bio gas”) AND (stove* OR cookstove* OR cooktop* OR cooking* OR “kitchen” OR “kitchens”)) OR (“lpg stove” or “lpg stoves” or “gas stove” or “gas stoves” or “electric stove” or “electric stoves” OR “ethanol stove” OR “ethanol stoves” OR “ethanol burner” OR “ethanol burners” or “gas burner” or “gas burners” or “gas cooktop” or “gas cooktops” OR “household energy” OR biodigester* OR “compressed biomass” OR briquette* OR “biomass pellet stove” OR “biomass pellet stoves”)) AND (“water boil time” OR “water boil times” OR “water boiling time” OR “water boiling times” OR “water boil test” OR “water boil tests” OR “water boiling test” OR “water boiling tests” OR “time factor” OR “time factors” OR “time savings” OR “times saved” OR “saved time” OR “time saved” OR “time spent” OR “time benefit” OR hour* OR minute* OR performance* OR consumption* OR “time saving”)

**Business Source Complete**

TI((((clean* AND fuel*) OR (clean* AND “energy”) OR (“biogas” OR “bio gas”) AND (stove* OR cookstove* OR cooktop* OR cooking* OR “kitchen” OR “kitchens”)) OR (“lpg stove” or “lpg stoves” or “gas stove” or “gas stoves” or “electric stove” or “electric stoves” OR “ethanol stove” OR “ethanol stoves” OR “ethanol burner” OR “ethanol burners” or “gas burner” or “gas burners” or “gas cooktop” or “gas cooktops” OR “household energy” OR biodigester* OR “compressed biomass” OR briquette* OR “biomass pellet stove” OR “biomass pellet stoves”)) AND (“water boil time” OR “water boil times” OR “water boiling time” OR “water boiling times” OR “water boil test” OR “water boil tests” OR “water boiling test” OR “water boiling tests” OR “time factor” OR “time factors” OR “time savings” OR “times saved” OR “saved time” OR “time saved” OR “time spent” OR “time benefit” OR hour* OR minute* OR performance* OR consumption* OR “time saving”)

**ABI/INFORM**

(((clean* AND fuel*) OR (clean* AND “energy”) OR (“biogas” OR “bio gas”) AND (stove* OR cookstove* OR cooktop* OR cooking* OR “kitchen” OR “kitchens”)) OR (“lpg stove” or “lpg stoves” or “gas stove” or “gas stoves” or “electric stove” or “electric stoves” OR “ethanol stove” OR “ethanol stoves” OR “ethanol burner” OR “ethanol burners” or “gas burner” or “gas burners” or “gas cooktop” or “gas cooktops” OR “household energy” OR biodigester* OR “compressed biomass” OR briquette* OR “biomass pellet stove” OR “biomass pellet stoves”)) AND (“water boil time” OR “water boil times” OR “water boiling time” OR “water boiling times” OR “water boil test” OR “water boil tests” OR “water boiling test” OR “water boiling tests” OR “time factor” OR “time factors” OR “time savings” OR “times saved” OR “saved time” OR “time saved” OR “time spent” OR “time benefit” OR hour* OR minute* OR performance* OR consumption* OR “time saving”)

## Appendix B

**Table A1:** Newcastle–Ottawa scale for cross-sectional studies (out of nine stars).

**Study**	**Selection**				Comparability	**Outcome**	
	Representativeness of the exposed (intervention) cohort	Sample size	Nonrespondents	Ascertainment of exposure		Assessment of outcome	Statistical test
Anderman et al.	*	*		*	**	*	*
Lewis et al.	*	*			**	*	

## Appendix C

**Table A2:** PRISMA 2009 checklist.

Section/Topic	#	Checklist Item	Reported on Page #
**Title**			
Title	1	Identify the report as a systematic review, meta-analysis, or both.	1
**Abstract**			
Structured summary	2	Provide a structured summary including, as applicable, background; objectives; data sources; study eligibility criteria, participants, and interventions; study appraisal and synthesis methods; results; limitations; conclusions and implications of key findings; systematic review registration number.	1
**Introduction**			
Rationale	3	Describe the rationale for the review in the context of what is already known.	2
Objectives	4	Provide an explicit statement of questions being addressed with reference to participants, interventions, comparisons, outcomes, and study design (PICOS).	2
**Methods**			
Protocol and registration	5	Indicate if a review protocol exists, if and where it can be accessed (e.g., web address), and, if available, provide registration information including registration number.	-
Eligibility criteria	6	Specify study characteristics (e.g., PICOS, length of follow-up) and report characteristics (e.g., years considered, language, publication status) used as criteria for eligibility, giving rationale.	2–3
Information sources	7	Describe all information sources (e.g., databases with dates of coverage, contact with study authors to identify additional studies) in the search and date last searched.	2–3
Search	8	Present full electronic search strategy for at least one database, including any limits used, such that it could be repeated.	2–3,10–13
Study selection	9	State the process for selecting studies (i.e., screening, eligibility, included in systematic review, and, if applicable, included in the meta-analysis).	3
Data collection process	10	Describe method of data extraction from reports (e.g., piloted forms, independently, in duplicate) and any processes for obtaining and confirming data from investigators.	3
Data items	11	List and define all variables for which data were sought (e.g., PICOS, funding sources) and any assumptions and simplifications made.	3–4
Risk of bias in individual studies	12	Describe methods used for assessing risk of bias of individual studies (including specification of whether this was done at the study or outcome level) and how this information is to be used in any data synthesis.	3
Summary measures	13	State the principal summary measures (e.g., risk ratio, difference in means).	3–4
Synthesis of results	14	Describe the methods of handling data and combining results of studies, if done, including measures of consistency (e.g., *I*^2^) for each meta-analysis.	-
Risk of bias across studies	15	Specify any assessment of risk of bias that may affect the cumulative evidence (e.g., publication bias, selective reporting within studies).	3
Additional analyses	16	Describe methods of additional analyses (e.g., sensitivity or subgroup analyses, metaregression), if done, indicating which were prespecified.	4
**Results**			
Study selection	17	Give numbers of studies screened, assessed for eligibility, and included in the review, with reasons for exclusions at each stage, ideally with a flow diagram.	4
Study characteristics	18	For each study, present characteristics for which data were extracted (e.g., study size, PICOS, follow-up period) and provide the citations.	4–5
Risk of bias within studies	19	Present data on risk of bias of each study and, if available, any outcome level assessment (see Item 12).	6
Results of individual studies	20	For all outcomes considered (benefits or harms), present, for each study: (a) Simple summary data for each intervention group and (b) effect estimates and confidence intervals, ideally with a forest plot.	5
Synthesis of results	21	Present results of each meta-analysis done, including confidence intervals and measures of consistency.	-
Risk of bias across studies	22	Present results of any assessment of risk of bias across studies (see Item 15).	6
Additional analysis	23	Give results of additional analyses, if done (e.g., sensitivity or subgroup analyses, meta-regression (see Item 16)).	6–7
**Discussion**			
Summary of evidence	24	Summarize the main findings, including the strength of evidence for each main outcome, and consider relevance to key groups (e.g., healthcare providers, users, and policy-makers).	7–8
Limitations	25	Discuss limitations at study and outcome level (e.g., risk of bias) and at review level (e.g., incomplete retrieval of identified research, reporting bias).	8
Conclusions	26	Provide a general interpretation of the results in the context of other evidence and implications for future research.	9
**Funding**			
Funding	27	Describe sources of funding for the systematic review and other support (e.g., supply of data) and the role of funders in the systematic review.	9
